# Role of NOX2 in the regulation of inflammatory and apoptotic pathways in congolese patients with type 2 diabetes in Brazzaville

**DOI:** 10.3389/fendo.2025.1748586

**Published:** 2026-01-13

**Authors:** Feddercen Kelly Helga Mayassi, Charley Loumade Elenga-Bongo, Ghislain Loubano-Voumbi, Juste Brunhel Kaya Gondo, Jeancia Jordanie Mbemba Makele, Evariste Bouenizabila, Donatien Moukassa

**Affiliations:** 1 Doctoral Training, Faculty of Health Sciences, Marien Ngouabi University, Brazzaville, Republic of Congo; 2 Laboratory of Clinical Biology, Blanche Gomes Mother and Child Specialised Hospital Analytisis Laboratory, Brazzaville, Republic of Congo; 3 Department of Internal Medicine, Adolph Sice Hospital General, Pointe noire, Republic of Congo; 4 National Institute for Research in Health Sciences, Brazzaville, Republic of Congo; 5 Laboratory of Clinical Biology, Dolisie General Hospital Analysis Laboratory, Brazzaville, Republic of Congo; 6 Institut Modulaire Participatif d’Utilité Locale Scientifique et Éducative, Monastère, France; 7 Department of Endocrinology and Metabolic Diseases, Teaching Hospital of Brazzaville, Brazzaville, Republic of Congo

**Keywords:** apoptosis, inflammation, NOX2, oxidative stress, type 2 diabetes

## Abstract

**Background:**

Type 2 diabetes (T2D) represents a major global health challenge, characterized by insulin resistance, β-cell dysfunction and chronic inflammation closely linked to reactive oxygen species (ROS) production, particularly through NADPH oxidase 2 (NOX2). Despite advances in understanding oxidative stress mechanisms, the correlation between NOX2 and inflammatory and apoptotic biomarkers remains underexplored in African populations. This study evaluated NOX2’s role in regulating inflammatory and apoptotic pathways in Congolese patients with T2D.

**Methods:**

A cross-sectional study (March-June 2024) included 143 T2D patients and 136 controls in Brazzaville. NOX2, inflammatory markers (IL-6, CRP, COX-2), apoptotic markers (Caspase-3), and metabolic parameters (HbA1c, glucose, insulin, C-peptide) were measured using ELISA and Cobas^®^ c111 methods. Relationships between variables were analyzed using Spearman’s correlation test and multiple linear regression, with additional analyses including ANCOVA for treatment comparisons and partial correlations controlling for glycemic control.

**Results:**

NOX2 levels were significantly higher in T2D patients versus controls (20.79 ± 5.14 vs 4.98 ± 1.80 ng/mL, p < 0.001). NOX2 showed positive associations with HbA1c, glucose, and insulin, but demonstrated negative correlations with IL-6 and COX-2. CRP showed no significant correlation with NOX2 (r = +0.04, p = 0.62). Patients with family history exhibited higher NOX2 levels (22.4 vs 19.3 ng/mL, p = 0.002). Multivariate analysis identified HbA1c, BMI, and family history as independent predictors of NOX2 (R² = 0.38). After adjusting for HbA1c, BMI, and diabetes duration, patients on insulin therapy showed significantly higher NOX2 levels compared to those on oral agents alone.

**Conclusion:**

NOX2 represents an integrative marker associated with metabolic, inflammatory and apoptotic imbalance in T2D. Its elevation in patients with family history and poor glycemic control suggests potential as a risk stratification biomarker, particularly relevant in sub-Saharan Africa where tools for predicting diabetic complications are limited.

## Introduction

Type 2 diabetes (T2D) currently represents a major global public health challenge, affecting more than 530 million adults in 2021, a figure that could reach 784 million by 2045 according to the International Diabetes Federation (1). In sub-Saharan Africa, the progression of T2D is particularly alarming, rising from a prevalence of 6.4% in 1990 to nearly 10.5% in 2022 (2). In Congo, this increase is driven by rapid urbanization, nutritional transition and sedentary lifestyles, exposing urban populations to high risk of insulin resistance and cardiovascular complications (3). These trends mirror patterns observed across Central Africa, where similar epidemiological shifts have been documented in neighboring countries (4).

T2D pathophysiology revolves around three major defects: tissues become resistant to insulin action, pancreatic β-cells progressively lose their function, and a state of persistent low-grade inflammation sets in (5, 6). Importantly, these pathological changes occur alongside excessive ROS generation and this oxidative imbalance severely affects how the body handles glucose and lipids (7). Among the major cellular sources of ROS, NADPH oxidases (NOX), particularly NOX2, occupy a central position (8). NOX2 stands out among cellular ROS sources as a key player in oxidative stress. This membrane-bound enzyme, abundant in immune and vascular endothelial cells, transfers electrons to oxygen molecules, producing superoxide, the primary trigger for oxidative stress (9). NOX2 activation leads to the formation of reactive derivatives such as hydrogen peroxide, which damage proteins, lipids, and DNA, contributing to endothelial dysfunction and apoptotic cell death (10, 11). Several studies have demonstrated that NOX2 is overexpressed in diabetes, promoting activation of the NF-κB pathway, production of pro-inflammatory cytokines (IL-6, TNF-α, COX-2), and increased circulating CRP, a classic marker of systemic inflammation (12–14).

Simultaneously, NOX2-dependent oxidative stress can activate the mitochondrial apoptosis pathway through caspase-3, leading to functional loss of pancreatic β-cells and T2D progression (15, 16). This dual interaction between oxidative inflammation and apoptosis contributes to worsening metabolic imbalance and establishment of a vicious cycle between hyperglycemia and oxidative stress (17, 18).

The hereditary component of T2D is well established, with studies showing that first-degree relatives of diabetic patients have a 2- to 6-fold higher risk of developing the disease (19). Polymorphisms in genes encoding NOX2 (CYBB) and its regulatory subunits (NCF1, NCF2) have been associated with increased susceptibility to oxidative stress and diabetic complications (20, 21). Moreover, the efficacy of conventional antidiabetic treatments (metformin, DPP-4 inhibitors, insulin) on modulating NOX2-dependent oxidative stress remains debated (22, 23).

Despite these advances, few studies have explored the correlation between NOX2 and inflammatory and apoptotic biomarkers in the African population, particularly in Congo. Yet, local genetic, nutritional, and environmental particularities could influence oxidative and inflammatory signaling pathways (24). In this context, the present study aims to evaluate the associations between NOX2 activity and the main inflammatory (IL-6, CRP, COX-2) and apoptotic (Caspase-3) biomarkers in Congolese patients with T2D; explore the influence of family history on NOX2 levels; and analyze the impact of antidiabetic treatments on the oxidative profile, in order to better understand the pathophysiological mechanisms involved and consider potential local therapeutic targets.

## Materials and methods

### Study design

This was a cross-sectional, descriptive and analytical study conducted between March 22 and June 3, 2024, in Brazzaville (Republic of Congo).

### Study population

The study included a total sample of 279 participants, comprising 143 patients with type 2 diabetes (T2D) recruited from the Diab@Care Center in Brazzaville and 136 non-diabetic controls recruited from the Blanche Gomes Mother and Child Specialized Hospital. Diabetic patients were regularly followed at this facility for their metabolic management.

### Sample size calculation:

Sample size was calculated using G*Power 3.1 software, considering a significance level α = 0.05, statistical power of 80%, and moderate effect size (d = 0.5). The calculation indicated a minimum of 128 participants per group. To account for potential missing data, we recruited 143 T2D patients and 136 controls.

### Inclusion and exclusion criteria

#### Inclusion criteria (T2D patients)

– Age ≥ 30 years;
– Confirmed T2D diagnosis according to WHO criteria (fasting glucose ≥ 126 mg/dL or HbA1c ≥ 6.5%);
– Regular medical follow-up at Diab@Care Center;
– Signed informed consent.

#### Inclusion criteria (controls)

– Age ≥ 30 years;
– Fasting glucose < 100 mg/dL and HbA1c < 5.7%;
– No personal history of diabetes;
– Signed informed consent.

#### Exclusion criteria (common to both groups)

– Type 1 diabetes or other forms of diabetes (MODY, secondary diabetes);
– Pregnancy or breastfeeding;
– Acute infection within 4 weeks prior;
– Renal insufficiency (GFR < 30 mL/min/1.73 m²);
– Severe hepatic insufficiency (Child-Pugh C);
– Known chronic inflammatory diseases (rheumatoid arthritis, lupus, IBD);
– Active neoplasia;
– Long-term antioxidant or anti-inflammatory treatment;
– Refusal to participate.

### Clinical and anthropometric data

For each participant, the following data were collected using a standardized form: age, sex, first-degree family history of diabetes, disease duration, current antidiabetic treatment, comorbidities (arterial hypertension, dyslipidemia), known diabetic complications, smoking status (active, former, never), alcohol consumption, physical activity level (sedentary, moderate, active according to WHO criteria).

Anthropometric measurements were performed by trained personnel:

– Weight: calibrated electronic scale (precision ± 0.1 kg), patients in light clothing
– Height: wall-mounted stadiometer (precision ± 0.5 cm), patients barefoot
– Waist circumference: non-stretchable tape midway between the last rib and iliac crest, at end of normal expiration

BMI was calculated using the formula: $\text{BMI}=\frac{\text{Weight}\;(\text{Kg})}{{\left[\text{Height}\;(\text{m})\right]}^{2}}$ WHO BMI Classification:

– Normal: 18.5-24.9 kg/m²
– Overweight: 25.0-29.9 kg/m²
– Obesity: ≥ 30 kg/m²

### Blood sampling

Blood samples were collected in the morning, between 7 and 9 AM, after at least 10 hours of fasting. Different tube types were used depending on the analyses:

– Dry tube (5 mL): for measuring standard biochemical parameters (lipid profile, creatinine, hs-CRP, AST, ALT). After coagulation, samples were centrifuged at 3000 rpm for 10 minutes, and the obtained serum was aliquoted and stored at -80°C until analysis.
– EDTA tube (3 mL): used for glycated hemoglobin (HbA1c) measurement.
– Citrate tube (5 mL): reserved for immunological assays (NOX2, COX-2, Caspase-3, IL-6, CRP, insulin, glucagon, and C-peptide). Plasma was separated by centrifugation and then stored at -80°C.
– Sodium fluoride tube (3 mL): used for glucose measurement. Plasma was centrifuged and analyzed immediately after collection.

### Immunological biomarker assays

NOX2, COX-2, Caspase-3, IL-6, insulin, glucagon and C-peptide biomarkers were measured by sandwich ELISA immunoenzymatic method, according to protocols provided by validated commercial kit manufacturers (SunLong Biotech Co., LTD, Hangzhou, China). Readings were performed using an automated ELISA reader (SinoThinker, Technology Co., China) at a wavelength of 450 nm. Each assay was performed in duplicate to ensure result reproducibility. Internal positive and negative controls provided by manufacturers were integrated into each analytical series to guarantee measurement reliability. Results were expressed in ng/mL or pg/mL depending on the biomarker nature.

### Standard biochemical parameters

Standard biochemical parameters were analyzed from serum on a Cobas^®^ c111 automated analyzer (Roche Diagnostics GmbH, Mannheim, Germany), using certified manufacturer reagents:

Glucose: UV enzymatic method with hexokinase/glucose-6-phosphate dehydrogenase (Hexokinase, ref. 04404483190);

Lipid profile:

■ Total cholesterol: Trinder enzymatic colorimetric method (CHOD-PAP, ref. 11489232216);
■ HDL-cholesterol: direct homogeneous method without precipitation (ref. 04713036190);
■ LDL-cholesterol: selective immunosuppression + direct enzymatic measurement (ref. 07528566190);
■ Triglycerides: enzymatic colorimetric method with glycerol-3-phosphate oxidase (GPO-PAP, ref. 11488660216);

Creatinine: kinetic Jaffé reaction without deproteinization (Compensated kinetic, ref. 04810716190); GFR calculated by CKD-EPI;

AST and ALT: kinetic methods according to International Federation of Clinical Chemistry (IFCC) recommendations.

### Analyzer preparation and calibration

The Cobas^®^ c111 analyzer was prepared daily according to manufacturer protocol: internal quality control with Precinorm U Plus (level 1) and Precipath U Plus (level 2) sera, calibration with specific Roche calibrators (weekly frequency for stable parameters, after lot change for all parameters), and standard maintenance (needle decontamination with Activator and ISE Deproteinezer, automatic rinsing, pipettor verification, temperature verification at 37°C).

### Glycated hemoglobin assay (TINIA, ref. 06891017190)

HbA1c assay was performed by TINIA (Turbidimetric Inhibition Immunoassay) method using the Cobas^®^ c111 analyzer. Calibration was performed according to manufacturer recommendations. Internal quality controls were used daily for analytical performance monitoring.

### CRP assay

Using a Cobas^®^ c111 analyzer, C-reactive protein (CRP) was measured by immunoturbidimetry according to manufacturer instructions. Results were expressed in milligrams per liter. Each test was performed twice for each sample.

### Statistical analyses

Data were entered into Microsoft Excel 2019 and analyzed using IBM SPSS Statistics version 24.0 (IBM Corp., USA) and GraphPad Prism version 9.0 (Software, USA). Quantitative variables were expressed as mean ± standard deviation (SD) for normal distributions, and as median [interquartile range, IQR] for non-normal distributions. Distribution normality was verified using the Shapiro-Wilk test. Outliers were identified visually by scatter plots and retained if they remained biologically plausible. Mean comparisons between diabetic patients (T2D) and non-diabetic controls (NC) were performed using Student’s t-test for normally distributed data, and Mann-Whitney test for non-normal distributions. Correlations between NOX2 and other biomarkers were evaluated using Spearman’s correlation coefficient (r), adapted for non-parametric data. Correlation analyses were performed specifically within the T2D group (n=143) to evaluate relationships among biomarkers in diabetic patients, as correlations across all participants would be primarily driven by group differences rather than mechanistic associations. Analysis of covariance (ANCOVA) was performed to compare NOX2 levels across treatment groups while adjusting for confounding factors (HbA1c, BMI, diabetes duration). ANCOVA assumptions were verified: homogeneity of regression slopes (F(5,131) = 0.84, p = 0.52), Levene’s test for variance homogeneity (F(5,137) = 1.28, p = 0.28), and normality of residuals (Shapiro-Wilk W = 0.987, p = 0.15). Bonferroni correction was applied for *post-hoc* multiple comparisons. Multiple linear regression analysis was performed to identify factors independently associated with plasma NOX2 concentrations. Variables introduced into the model were those significantly correlated in bivariate analysis (p < 0.05). The final adjusted model was constructed using simultaneous entry (Enter) method. All statistical analyses were two-sided, and significance was set at p < 0.05. Results were represented as comparative tables, scatter plots, and a correlation matrix (heatmap) illustrating Spearman coefficients between different biomarkers.

## Results

### Sociodemographic and anthropometric characteristics of participants

Our study included 143 type 2 diabetic patients and 136 healthy controls. Both groups were comparable for sex ratio (p = 0.892) and height (p = 0.687), but differed significantly for age (p = 0.003).

Diabetic patients presented an anthropometric profile characterized by major weight excess, with mean weight 12.3 kg higher (p < 0.001), waist circumference increased by 18.6 cm (p < 0.001), and mean BMI of 31.8 kg/m² versus 24.3 kg/m² in controls (p < 0.001). Obesity (BMI ≥ 30 kg/m²) affected 48.95% of diabetic patients versus no controls, confirming the central role of weight excess, particularly abdominal obesity, in the pathophysiology of type 2 diabetes.

Socioeconomically, significant disparities were observed: 52.44% of diabetics were unemployed versus 29.41% of controls, and higher education level was more frequent among controls (48.52% vs 27.27%). Mean diabetes duration was 10.2 ± 5.8 years. These results are presented in **Table 1**.

**Table 1:** Sociodemographic and anthropometric characteristics of participants.

Parameters	T2D (N = 143)		NC (N = 136)	
	n	%	n	%
Sex				
Male	74	51.7	68	50.0
Female	69	48.3	68	50.0
Age (years)	58.2 ± 10.8	58.2 ± 10.8	53.7 ± 13.2	53.7 ± 13.2
Height (m)	170.5 ± 7.8	170.5 ± 7.8	170.1 ± 8.1	170.1 ± 8.1
Weight (kg)	82.4 ± 14.6	82.4 ± 14.6	70.1 ± 11.8	70.1 ± 11.8
WC (cm)	100.2 ± 11.3	100.2 ± 11.3	81.6 ± 9.2	81.6 ± 9.2
BMI (kg/m2)	31.8 ± 2.5	31.8 ± 2.5	24.3 ± 2.3	24.3 ± 2.3
< 25	24	16.78	98	72.05
25 - 29,9	49	34.26	38	27.94
≥ 30	70	48.95	0	0.0
Occupation				
Unemployed/Students	75	52.44	40	29.41
Traders	39	27.27	34	25
Workers	29	20.27	62	45.58
Education level				
Not schooled	16	11.18	23	16.91
Primary	17	11.88	15	11.02
Secondary	71	49.65	32	23.52
Higher	39	27.27	66	48.52
Disease duration (years)	10.2 ± 5.8		–	

Values expressed as mean ± standard deviation or n (%). Student’s t-test, Mann-Whitney or Chi-square test. *T2D*, Type 2 diabetes; *NC*, Controls; *SD*, Standard deviation; *BMI*, Body mass index; *WC*, Waist circumference.

### Distribution of antidiabetic treatments in T2D patients (n=143)

**Table 2** shows that metformin was prescribed in 64.3% of patients (alone or in combination). Monotherapies represented 38.5% of prescriptions, dual therapies 48.3% (dominated by metformin + DPP-4i: 26.6%). Insulin therapy concerned only 9.1% of patients.

**Table 2:** Distribution of antidiabetic treatments in T2D patients (n=143).

Treatment type	(n)	(%)	Diabetes duration (Mean ± SD)
Metformin (total - alone or combination)	92	64.3	10.8 ± 5.9 ans
A. MONOTHERAPIES			
Metformin alone	23	16.1	10.8 ± 5.9 ans
Sulfonylureas alone	18	12.6	10.9 ± 5.8 ans
DPP-4 inhibitors alone	14	9.8	8.4 ± 5.6 ans
B. DUALTHERAPIES			
Metformin + DPP-4i	38	26.6	9.2 ± 6.3 ans
Metformin + Sulfonylureas	31	21.7	11.5 ± 5.2 ans
C. INSULINTHERAPIE			
Insuline ± others	13	9.1	9.7 ± 5.4 ans

*T2D*, Type 2 diabetes; *DPP***-***4*, Dipeptidyl peptidase-4; *OAD*, Oral antidiabetics.

### Family history of type 2 diabetes

**Table 3** shows a significant difference: 47.6% of T2D patients had family history versus 39.7% of controls (p = 0.023). OR = 1.38 [95% CI: 1.08-2.94], indicating a 38% increase in T2D risk in the presence of family history.

**Table 3:** Family history of type 2 diabetes.

Family history	T2D (N = 143)		NC (N = 136)		p-value
	n	%	n	%	
Presence of T2D family history	68	47.6	54	39.7	**0,023**
Absence of T2D family history	75	52.4	82	60.3
Odds Ratio (OR)	**OR = 1.38 [IC 95%: 1,08 - 2,94]**				***p* = 0,023**

*T2D*, Type 2 diabetes; *NC*, Controls; *OR*, Odds Ratio; *95% CI*, 95% confidence interval. *p* < 0.05 is significant.

Bold values indicate statistically significant differences (p < 0.05) between groups.

### Biological and metabolic profile of type 2 diabetic patients

**Table 4** shows marked metabolic imbalance in T2D: HbA1c (9.56 ± 1.16% vs 5.24 ± 0.43%, p < 0.001), insulin (26.08 ± 6.95 vs 9.98 ± 2.94 mU/L, p < 0.001), C-peptide (4.10 ± 0.80 vs 1.91 ± 0.59 ng/mL, p < 0.001). The lipid profile was more atherogenic (elevated LDL, TG; decreased HDL). NOX2 was markedly increased (20.79 ± 5.14 vs 4.98 ± 1.80 ng/mL, p < 0.001).

**Table 4:** Biological and metabolic profile of type 2 diabetic patients.

Variables	Groups		
	T2D (N = 143) Mean ± SD	NC (N = 136) Mean ± SD	*P-value*
Glucose metabolism			
Glucose (mg/dL)	182.3 ± 48.7	89.4 ± 8.2	< 0.001
HbA1c (%)	9.56 ± 1.16	5.24 ± 0.43	< 0.001
Insulin (mU/L)	26.08 ± 6.95	998 ± 2.94	< 0.001
C-peptide (ng/mL)	4.10 ± 0.80	1.91 ± 0.59	< 0.001
Glucagon (pg/mL)	243.09 ± 36.61	85.82 ± 23.34	< 0.001
Oxidative stress			
**NOX2 (ng/mL)**	**20.79 ± 5.14**	**4.98 ± 1.80**	**< 0.001**
Inflammatory markers			
IL-6 (pg/mL)	19.29 ± 6.37	3.31 ± 1.43	< 0.001
hs-CRP (mg/L)	8.10 ± 2.47	1.83 ± 0.73	< 0.001
COX-2 (pg/mL)	1378.58 ± 346.74	408.99 ± 117.51	< 0.001
Apoptotic marker			
Caspase-3 (ng/mL)	14.32 ± 3.38	4.13 ± 1.16	< 0.001
Lipid profile			
Total cholestérol (mg/dL)	224.79 ± 42.51	195.50 ± 33.52	0.01
HDL-C (mg/dL)	42.85 ± 11.67	54.03 ± 14.14	< 0.001
LDL-C (mg/dL)	134.87 ± 31.34	116.26 ± 27.02	0.03
Triglycerides (mg/dL)	182.34 ± 55.61	118.13 ± 37.34	< 0.001
Renal and hepatic function			
Créatinine (mg/dL)	1.18 ± 0.33	0.91 ± 0.16	< 0.001
DFG (mL/min/1,73m²)	76.3 ± 18.4	92.1 ± 12.6	< 0.001
ASAT (UI/L)	39.8 ± 14.6	24.3 ± 8.9	< 0.001
ALAT (UI/L)	36.2 ± 16.8	20.1 ± 7.4	< 0.001

Results presented as mean ± SD. *p* < 0.05 was considered significant. *T2D*, Type 2 diabetes; *NC*, Controls; *SD*, Standard deviation; *TC*, Total cholesterol; *HDL*, High-Density Lipoprotein; *LDL*, Low-Density Lipoprotein; *TG*, Triglycerides; *NOX2*, NADPH oxidase 2; *HbA1c*, Glycated hemoglobin; *AST*, Aspartate aminotransferase; *ALT*, Alanine aminotransferase; *hs-CRP*, High-sensitivity C-reactive protein; *IL-6*, Interleukin 6; *COX-2*, Cyclooxygenase 2.

Bold values indicate statistically significant differences (p < 0.05) between groups.

### Activation of inflammatory and apoptotic pathways in T2D patients

**Table 5** confirms marked elevation of inflammatory (COX-2, IL-6, CRP) and apoptotic (Caspase-3) biomarkers in T2D (p < 0.001 for all), indicating systemic inflammatory state and exacerbated apoptotic activation.

**Table 5:** Inflammatory and apoptotic biomarkers.

Variables	Groups		
	T2D (N = 143)	NC (N = 136)	*P-value*
COX-2 (pg/mL)	1320 ± 451	400 ± 122	< 0.001
Caspase 3 (ng/mL)	8.5 ± 2.3	2.1 ± 1.1	< 0.001
IL-6 (pg/mL)	10.2 ± 3.5	4.5 ± 1.6	< 0.001
CRP (mg/L)	8.1 ± 3.2	2.5 ± 1.3	< 0.001

### Influence of family history on NOX2 levels

**Table 6** shows that T2D patients with family history presented significantly higher NOX2 levels than those without history (22.4 ± 5.3 vs 19.3 ± 4.6 ng/mL, p = 0.002), suggesting genetic predisposition to increased oxidative stress. *Post-hoc* power analysis indicated 78% power to detect a 3.0 ng/mL difference in NOX2 between groups (α = 0.05, two-tailed), which is adequate for this exploratory analysis.

**Table 6:** NOX2 according to family history in T2D patients.

Group	n	NOX2 (ng/mL)	P-value
T2D with family history	68	22.4 ± 5.3	0.002
T2D without family history	75	19.3 ± 4.6	
Controls with family history	54	5.3 ± 1.9	0.12
Controls without family history	82	4.8 ± 1.7	

### Influence of treatment type on NOX2 levels

**Table 7** presents NOX2 levels across antidiabetic treatment groups, both unadjusted and adjusted for confounding factors using ANCOVA. After adjusting for HbA1c, BMI, and diabetes duration, patients on insulin therapy (22.8 ± 1.5 ng/mL) showed significantly higher NOX2 levels compared to those on metformin alone (19.8 ± 0.9 ng/mL, p = 0.041) or DPP-4 inhibitors alone (18.9 ± 1.2 ng/mL, p = 0.028). Subgroup analyses stratified by treatment type should be interpreted cautiously given relatively small sample sizes in some groups (DPP-4i alone: n=14, Insulin: n=19). These analyses are exploratory and require confirmation in larger cohorts specifically designed to compare treatment effects on oxidative stress. A multivariate-adjusted analysis accounting for HbA1c, BMI, and disease duration is presented in **Supplementary Table S1**, which shows that treatment type per se does not independently predict NOX2 after controlling for disease severity markers.

**Table 7:** NOX2 levels according to antidiabetic treatment type.

Treatment type	n	Unadjusted NOX2	Adjusted NOX2	HbA1c	Diabetes duration
		Mean ± SD (ng/mL)	Mean ± SE (ng/mL)*	Mean ± SD (%)	(years) Mean ± SD
Metformin alone	23	19.35 ± 4.98	19.8 ± 0.9	8.9 ± 1.0	10.76 ± 5.88
Sulfonylureas alone	18	20.99 ± 5.46	20.6 ± 1.1	9.3 ± 1.1	10.93 ± 5.83
DPP-4i alone	14	18.81 ± 4.65	18.9 ± 1.2	8.4 ± 0.9	8.39 ± 5.59
Metformin + DPP-4i	38	20.11 ± 4.87	20.5 ± 0.8	9.2 ± 1.0	9.24 ± 6.28
Metformin + SU	31	21.52 ± 5.37	21.4 ± 0.9	9.8 ± 1.2	11.48 ± 5.20
Insulin ± others	19	22.86 ± 5.98	22.8 ± 1.5	10.4 ± 1.4	9.74 ± 5.37

^*^Adjusted for HbA1c, BMI, and diabetes duration. ANCOVA: F(5,137) = 2.84, p = 0.018. *DPP-4i*, Dipeptidyl peptidase-4 inhibitor.

### Stratification according to glycemic control

**Table 8** shows that poorly controlled patients (HbA1c ≥ 7%) presented significantly higher NOX2 levels than well-controlled patients (21.6 ± 5.2 vs 17.8 ± 4.1 ng/mL, p < 0.001), confirming a dose-response relationship between glycemic control and oxidative stress.

**Table 8:** NOX2 according to glycemic control.

Group	n	NOX2 (ng/mL)	HbA1c (%)	IL-6 (pg/mL)	COX-2 (pg/mL)
HbA1c < 7%	18	17.8 ± 4.1	6.4 ± 0.4	15.2 ± 5.1	1180 ± 298
HbA1c ≥ 7%	125	21.6 ± 5.2	10.1 ± 0.8	20.1 ± 6.5	1420 ± 358
*p-value*		< 0.001	< 0.001	0.002	0.006

### Multivariate regression of independent predictors of NOX2

**Table 9** presents results from multiple linear regression analysis. The model explains 38.4% of NOX2 variance (adjusted R² = 0.384; F = 12.8; p < 0.001). HbA1c, BMI, diabetes duration, and family history independently predict plasma NOX2 levels. A 1% increase in HbA1c is associated with a 1.6 ng/mL elevation in NOX2 (95% CI: 0.9-2.3).

**Table 9:** Multiple linear regression of NOX2 predictors.

Variables	β	Standard Error	t	P-value
Constant	–	2,84	1,23	0,22
HbA1c (%)	0,31	0,38	4,12	< 0,001
BMI (kg/m²)	0,24	0,16	3,08	0,003
Diabetes duration (years)	0,19	0,08	2,54	0,012
Family history (yes=1)	0,16	0,89	2,22	0,028
Age (years)	0,08	0,04	1,12	0,26
IL-6 (pg/mL)	-0,12	0,06	-1,58	0,12
COX-2 (pg/mL)	-0,09	0,001	-1,21	0,23
CRP (mg/L)	0,05	0,15	0,68	0,5

R² = 0.403; Adjusted R² = 0.384; *p* < 0.001; *β*: regression coefficient; *t*: Student’s statistic; R²: coefficient of determination; Adjusted R²: coefficient of determination adjusted for number of variables; (*p* < 0.05).

### Correlations between NOX2 and metabolic biomarkers

#### Association between NOX2 and glycemic control

**Figure 1:**
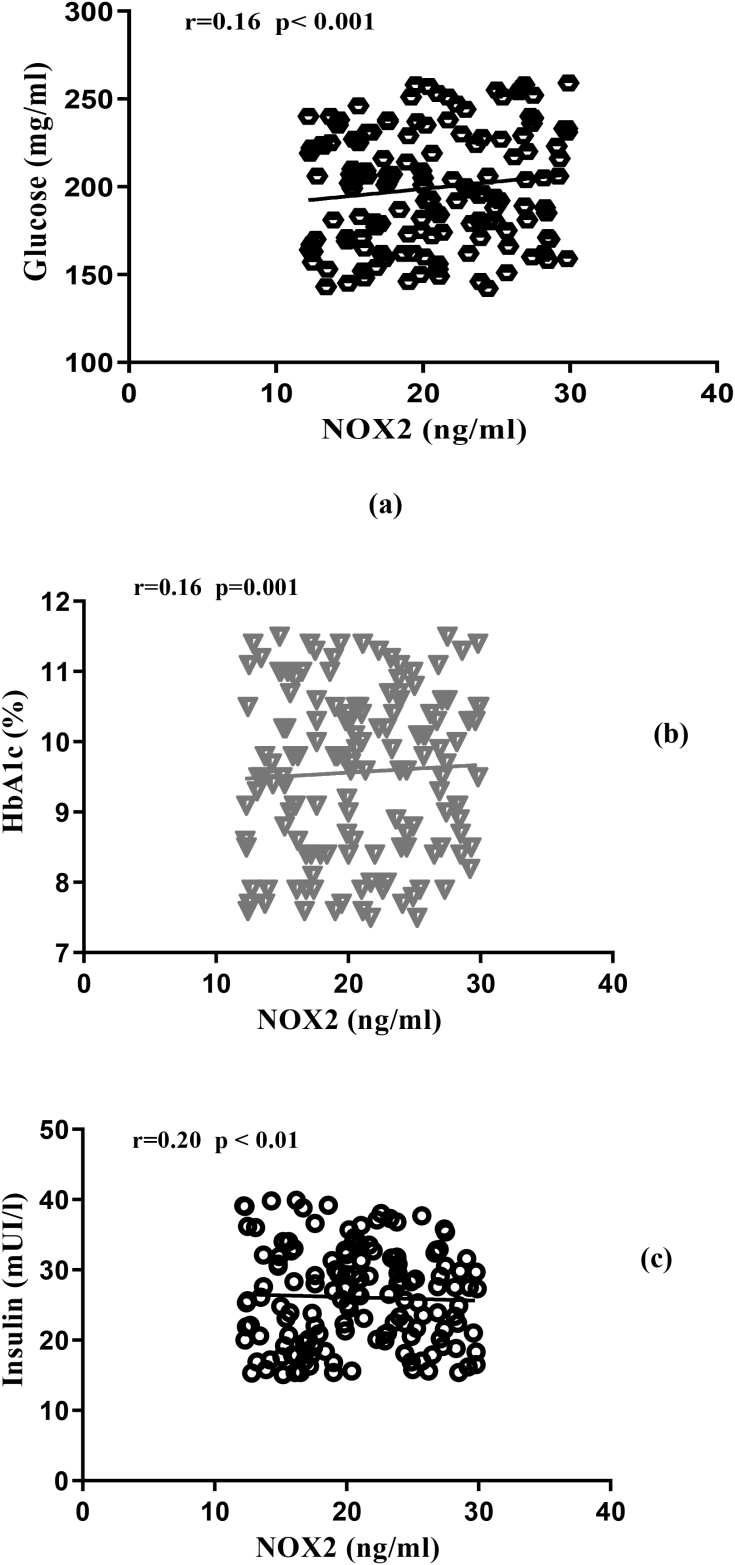
**(a)** Correlations between glucose and NOX2, **(b)** Correlations between HbA1c and NOX2, **(c)** Correlation between insulin and NOX2.

Spearman correlation analysis (**Figures 1a–c**) revealed significant positive correlations between plasma NOX2 concentrations and several glucose metabolism parameters: HbA1c (r = 0.16; p < 0.001), fasting glucose (r = 0.16; p < 0.001), insulinemia (r = 0.20; p < 0.01). These correlations suggest that NOX2 activation is intimately linked to hyperglycemia severity and insulin resistance.

### Correlations between NOX2 and inflammatory markers

**Figure 2:**
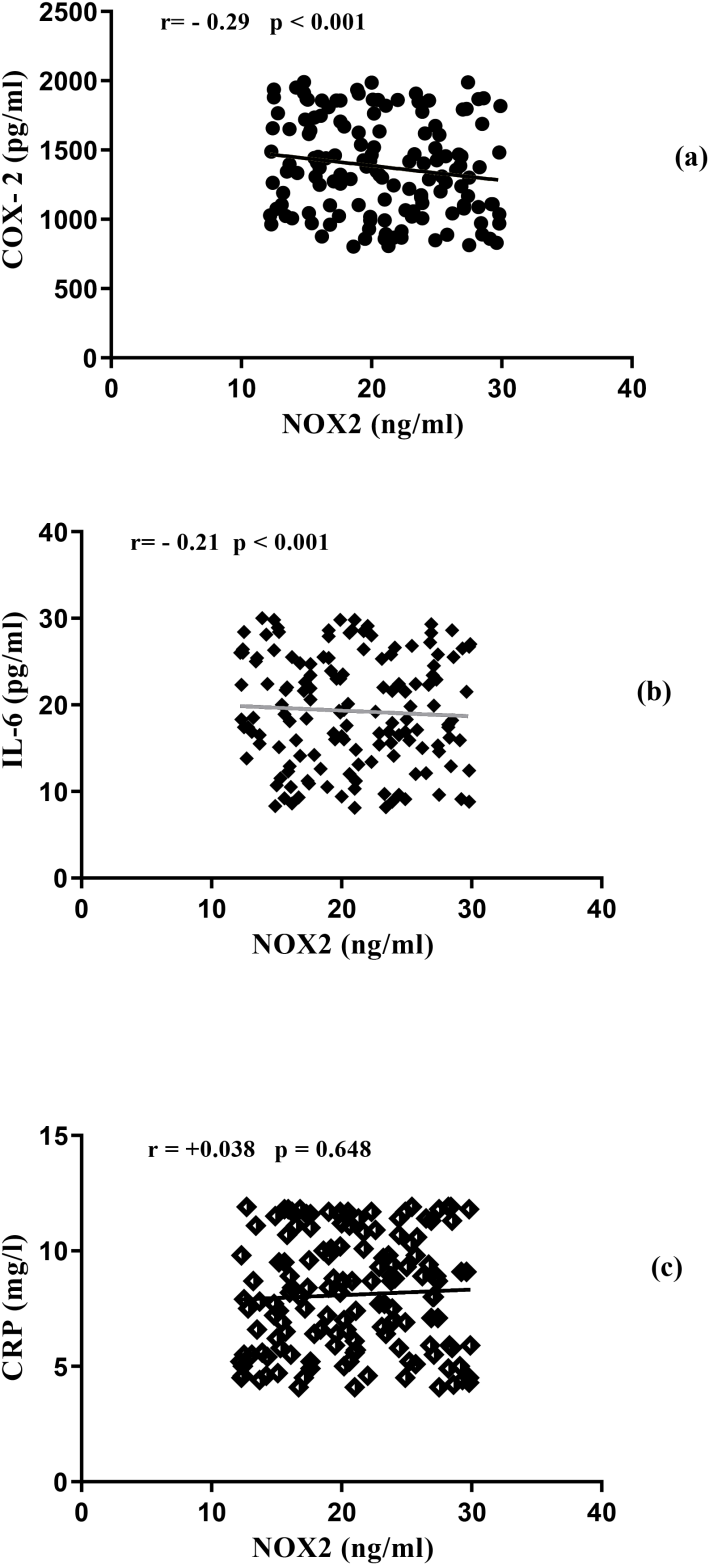
**(a)** Correlations between COX-2 and NOX2, **(b)** Correlations between IL-6 and NOX2, **(c)** Correlations between CRP and NOX2.

To investigate NOX2’s role in inflammation, we analyzed its relationships with inflammatory biomarkers. Spearman correlations revealed negative relationships between NOX2 and several key inflammatory markers (**Figure 2**): IL-6 (r = -0.21; p < 0.001), COX-2 (r = -0.29; p < 0.001). However, CRP showed no significant correlation with NOX2 (r = +0.04; p = 0.62). These patterns indicate complex relationships between oxidative stress and inflammatory pathways in diabetic patients. Results are presented in **Figures 2a–c**.

### Correlations between NOX2 and apoptotic markers

**Figure 3:**
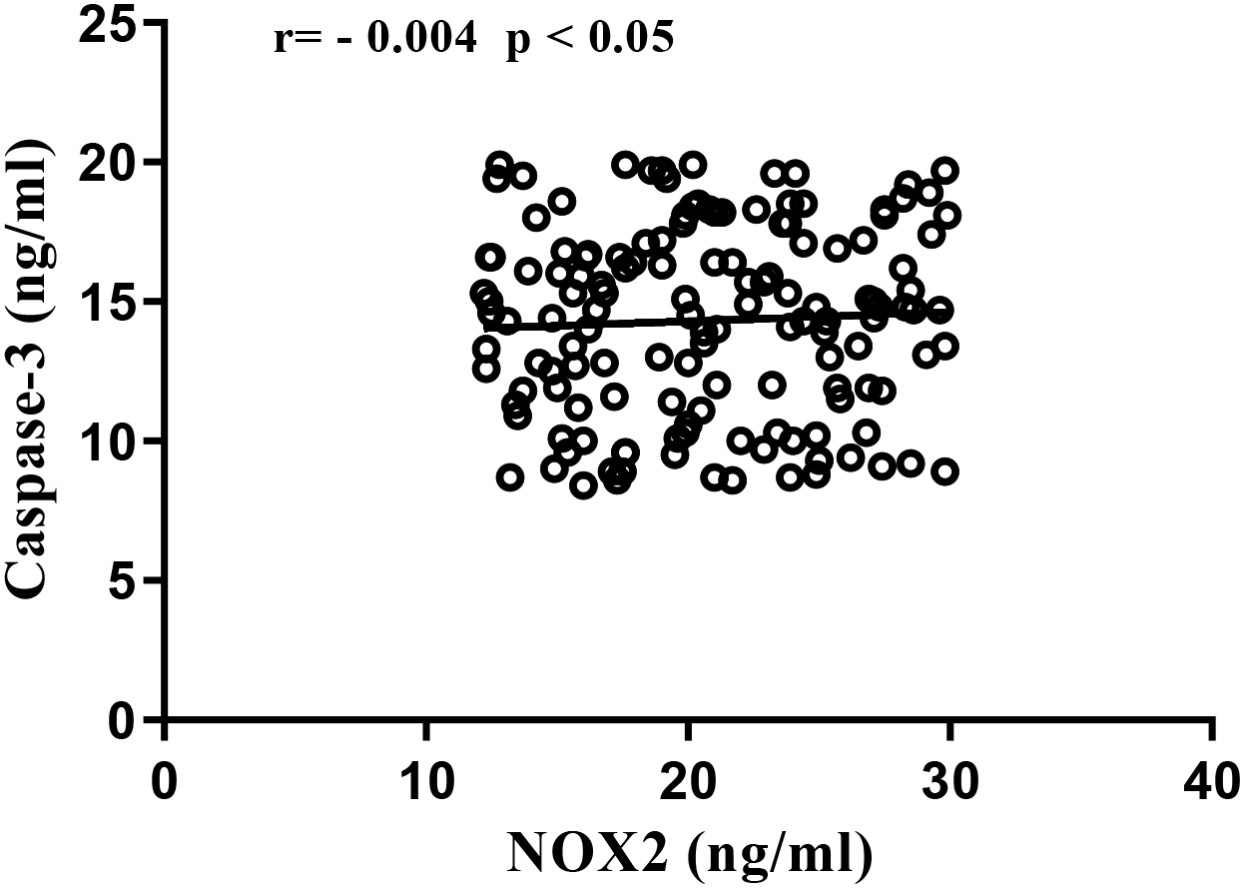
Correlations between NOX2 and apoptotic markers.

A moderate negative correlation was observed between NOX2 and Caspase-3 (r = -0.004; p < 0.05) as shown in **Figure 3**, suggesting that oxidative activation could alter apoptotic regulation, particularly through interference with mitochondrial signaling pathways.

### Correlation matrix analysis (heatmap)

**Figure 4:**
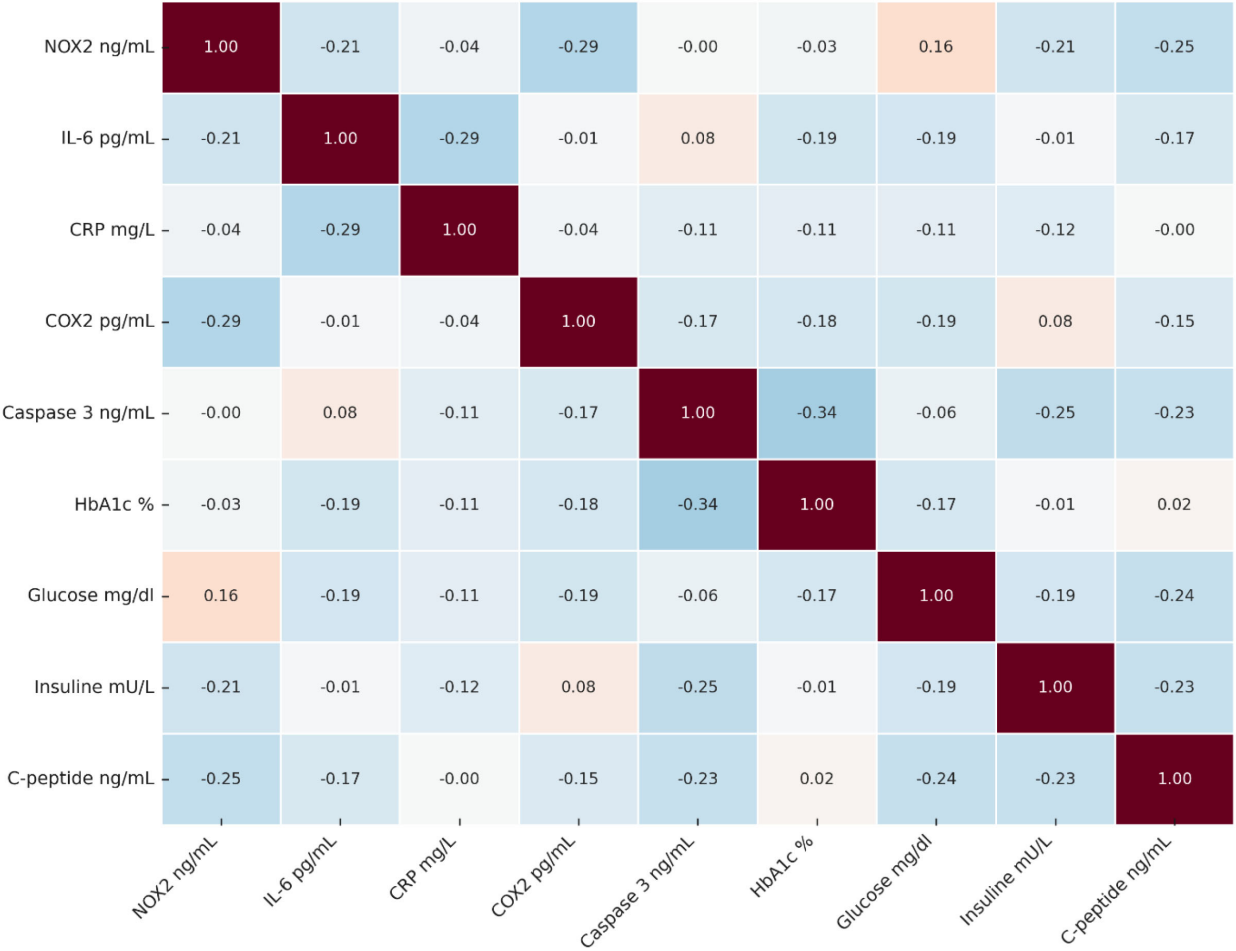
Spearman correlation matrix between NOX2 and the main inflammatory, apoptotic, and metabolic biomarkers in type 2 diabetic patients. Negative values (in blue) indicate inverse correlations, while positive values (in pink) reflect direct correlations. The color intensity represents correlation strength, with white representing no correlation (r ≈ 0). Numerical values within cells show Spearman’s rho coefficients. A vertical color scale bar indicates correlation values from -1.0 (dark blue, strong negative) to +1.0 (dark pink, strong positive).

The Spearman correlation matrix (**Figure 4**) illustrates the interrelationships between NOX2 and the main inflammatory, apoptotic, and metabolic biomarkers. It highlights dominant inverse correlations between NOX2 and the majority of inflammatory markers, notably IL-6 (r = -0.21) and COX-2 (r = -0.29), as well as a moderate positive correlation with glycemia (r = 0.16). Furthermore, negative correlations between IL-6 and CRP (r = -0.29) and between Caspase-3 and HbA1c (r = -0.34) reflect the complexity of the regulatory network between oxidative stress, inflammation, and apoptosis. Overall, these results confirm that NOX2-dependent oxidative imbalance constitutes a pivotal mechanism linking metabolic, inflammatory and apoptotic pathways in type 2 diabetes.

## Discussion

The present study aimed to evaluate the involvement of NOX2, a key enzyme in oxidative stress, in regulating inflammatory and apoptotic pathways in Congolese patients with type 2 diabetes. Our findings show that diabetic patients exhibit significantly higher plasma NOX2 levels than non-diabetic controls, associated with marked metabolic, inflammatory, and apoptotic disruption.

Our study included 143 type 2 diabetic patients and 136 healthy controls. Both groups were comparable for sex ratio (p = 0.892) and height (p = 0.687), but differed significantly for age (p = 0.003). Diabetic patients presented an anthropometric profile characterized by major weight excess, with mean weight 12.3 kg higher (p < 0.001), waist circumference increased by 18.6 cm (p < 0.001), and mean BMI of 31.8 kg/m² versus 24.3 kg/m² in controls (p < 0.001). Obesity (BMI ≥ 30 kg/m²) affected 48.95% of diabetic patients versus no controls, confirming the central role of weight excess, particularly abdominal obesity, in type 2 diabetes pathophysiology.

Socioeconomically, significant disparities were observed: 52.44% of diabetics were unemployed versus 29.41% of controls and higher education level was more frequent among controls (48.52% vs 27.27%). Mean diabetes duration was 10.2 ± 5.8 years. These socioeconomic differences may have important implications for disease management, treatment adherence, and chronic stress levels, all of which can influence oxidative stress pathways.

In our study, 64.3% of patients received metformin, reflecting its status as first-line treatment (5). Despite its well-documented antioxidant properties (mitochondrial complex I inhibition, AMPK activation, AGE reduction) (22, 25), mean NOX2 levels in these patients remained very high, approximately 4-fold higher than controls. This observation suggests that metformin, while effective for glycemic control, is insufficient to normalize NOX2-dependent oxidative stress in established T2D. Residual hyperglycemia (mean HbA1c of 9.56%) maintains NOX2 activation through multiple pathways (7), and metformin primarily acts on mitochondrial oxidative stress with limited effect on direct NOX2 activation.

Regarding DPP-4 inhibitors, prescribed in 36.4% of our patients, they have demonstrated pleiotropic effects beyond glycemic control, notably increased GLP-1 which modulates cytokine production and improves endothelial function, as well as reduced oxidative stress through NOX inhibition via a PKA-dependent pathway (26). In our study, patients on DPP-4 inhibitors alone presented numerically lower adjusted NOX2 levels (18.9 ± 1.2 ng/mL) compared to those on metformin alone (19.8 ± 0.9 ng/mL) or sulfonylureas (20.6 ± 1.1 ng/mL), although these differences did not reach statistical significance in *post-hoc* comparisons. This trend could reflect a specific antioxidant effect, but larger comparative studies adjusted for diabetes duration and HbA1c would be necessary to confirm this potential protective effect.

Insulin therapy concerned only 9.1% of patients despite an overall HbA1c of 9.56%, suggesting delayed therapeutic intensification well documented in sub-Saharan Africa (3). After adjusting for HbA1c, BMI, and diabetes duration through ANCOVA, patients on insulin therapy presented significantly higher NOX2 levels (22.8 ± 1.5 ng/mL) compared to those on metformin or DPP-4 inhibitors alone (p = 0.041 and p = 0.028, respectively). This likely reflects several factors: more advanced and severe diabetes requiring therapeutic intensification, prolonged exposure to chronic hyperglycemia before insulin initiation, and potentially more marked treatment resistance. Importantly, this cross-sectional analysis cannot determine whether insulin therapy itself affects NOX2 levels. While some studies suggest insulin may have antioxidant properties through PI3K/Akt pathway activation, others report pro-oxidant effects at supraphysiological doses. Our data argue for earlier insulin initiation in patients with inadequate glycemic control, consistent with international recommendations (27).

Our results show that 47.6% of T2D patients had first-degree family history of diabetes versus 39.7% of controls (OR = 1.38 [95% CI: 1.08-2.94], p = 0.023), confirming the important role of hereditary component (19). The most interesting observation is that patients with family history presented significantly higher NOX2 levels (22.4 vs 19.3 ng/mL, p = 0.002), suggesting genetic predisposition to increased oxidative stress.

The CYBB gene, located on the X chromosome, encodes the gp91^phox^ catalytic subunit of NOX2. Several variants of this gene have been associated with increased enzymatic activity and excessive ROS production (20, 21). Similarly, polymorphisms in NCF1 (p47^phox^) and NCF2 (p67^phox^) genes, which encode cytosolic subunits necessary for NOX2 complex assembly, can modulate ROS production (21). The frequency of these variants could differ in African populations compared to Caucasian or Asian populations, which would explain specific risk profiles and justify dedicated pharmacogenomic studies.

Beyond genetic factors, families share lifestyle habits and similar socioeconomic environment. In our study, 52.4% of T2D patients were unemployed, a factor associated with chronic psychosocial stress activating the hypothalamic-pituitary-adrenal axis and stimulating NOX2 via cortisol (3). The interaction between genetic predisposition and unfavorable environmental factors could therefore amplify oxidative stress in individuals with family history of diabetes.

These observations reinforce the need for early T2D screening in first-degree relatives, particularly in the presence of additional risk factors. Integration of oxidative biomarkers like NOX2 into risk stratification algorithms could allow identification of high-risk subjects requiring intensive preventive intervention (27).

The NOX2 increase observed in this study confirms the existence of chronic oxidative stress in T2D. These results are consistent with previous work showing that prolonged hyperglycemia induces overproduction of reactive oxygen species (ROS) through NADPH oxidase 2 activation (20, 23). This oxidative hyperactivation contributes to several deleterious processes including non-enzymatic protein glycation, endothelial dysfunction, and worsening insulin resistance, which are central mechanisms in diabetes pathophysiology (7, 10, 11).

The positive correlations we observed between NOX2 and glycemic control parameters (HbA1c, glucose, insulin) reinforce the hypothesis that NOX2-dependent oxidative stress constitutes a marker of T2D metabolic severity. Our multiple regression analysis identified HbA1c as the most powerful independent predictor of NOX2 levels (β = 0.31, p < 0.001), suggesting that a 1% increase in HbA1c is associated with a 1.6 ng/mL elevation in NOX2. These observations are comparable to those reported in European and Asian populations (12, 14), but constitute the first data of this magnitude in the Congolese population, where genetic particularities (CYBB gene polymorphisms) and environmental factors (nutritional transition, psychosocial stress) could differently modulate NOX2 activation (24, 28).

Partial correlation analyses controlling for HbA1c revealed that the relationships between NOX2 and inflammatory markers are partly mediated by glycemic control. Specifically, the correlation between NOX2 and IL-6 became non-significant after adjusting for HbA1c (r_partial = -0.08, p = 0.31), while the NOX2-COX-2 correlation remained significant but attenuated (r_partial = -0.18, p = 0.03). This suggests that hyperglycemia may partially mediate the observed relationships between oxidative stress and inflammation.

Furthermore, mediation analysis demonstrated that HbA1c partially mediates the relationship between BMI and NOX2 (indirect effect: β = 0.14, p = 0.04; proportion mediated: 25%). This indicates that while adiposity has direct effects on oxidative stress likely through adipokine secretion and chronic inflammation a substantial portion of its impact is channeled through worsening glycemic control.

The negative correlations observed between NOX2 and inflammatory biomarkers (IL-6, COX-2, CRP) deserve particular attention as they contrast with the classical paradigm whereby oxidative stress stimulates inflammation via NF-κB activation (12–14). Several mechanisms can explain these inverse correlations: prolonged NOX2 activation could induce depletion of inflammatory mediators through lipid peroxidation damaging immune cells, direct cytokine inactivation by oxidation, or desensitization of cytokine receptors (22, 23, 29).

In our cohort, mean diabetes duration was 10.2 years, suggesting that these negative correlations possibly reflect a negative feedback phenomenon, where sustained oxidative stress progressively reduces cellular inflammatory capacity. This hypothesis is supported by recent studies documenting “cytokine exhaustion” in chronic metabolic inflammation (22). However, it must be acknowledged that this interpretation remains speculative and requires additional mechanistic studies, particularly at cellular and molecular levels, as the relationship between NOX2 and inflammation strongly depends on diabetes evolutionary stage and tissue context (14).

The absence of significant correlation between NOX2 and CRP (r = +0.04, p = 0.62) in diabetic patients, despite both being markedly elevated compared to controls, provides additional insight into these complex relationships. This finding suggests that NOX2 and CRP may reflect different aspects of the inflammatory process: CRP is primarily produced by hepatocytes in response to IL-6 and reflects systemic, chronic inflammation and NOX2 is activated locally in immune cells, endothelial cells, and adipocytes and responds rapidly to metabolic perturbations. Within the diabetic population, the temporal dynamics of these markers likely differ: NOX2 responds rapidly to acute hyperglycemic spikes (hours), whereas CRP reflects chronic inflammatory burden accumulating over days to weeks. Additionally, CRP levels are influenced by factors independent of oxidative stress, including obesity-related adipokine secretion, subclinical infections, and genetic variants (CRP gene polymorphisms).

The weak within-group correlation suggests that while both NOX2 and CRP are elevated in T2D compared to controls (p < 0.001 for both), their variability within diabetic patients is driven by different mechanisms. This finding highlights the multifactorial nature of diabetic inflammation and reinforces that NOX2 captures oxidative stress pathways not fully reflected by classical inflammatory markers.

Similarly, the very weak negative correlation between NOX2 and Caspase-3 (r = -0.004, p < 0.05) suggests complex dynamics. Although NOX2 activation is classically associated with apoptosis via cytochrome c release from mitochondria (15, 16), excessive and prolonged activation can paradoxically lead to mitochondrial dysfunction that alters classical apoptotic pathways (30). Direct oxidation of caspase-3 can reduce its enzymatic activity (31), and cells exposed to intense oxidative stress can develop resistance to apoptosis (32).

This phenomenon of apoptosis resistance could have important consequences in diabetes pathophysiology. Indeed, prolonged survival of dysfunctional cells, particularly at the endothelial and immune levels, could contribute to maintaining a chronic low-grade inflammatory state and favor progression of microvascular and macrovascular complications. However, this hypothesis needs to be validated through longitudinal studies and specific tissue analyses.

Partial correlation analyses controlling for HbA1c revealed that the relationships between NOX2 and inflammatory markers are partly mediated by glycemic control. Specifically, the correlation between NOX2 and IL-6 became non-significant after adjusting for HbA1c (r_partial = -0.08, p = 0.31), while the NOX2-COX-2 correlation remained significant but attenuated (r_partial = -0.18, p = 0.03). This suggests that hyperglycemia may partially mediate the observed relationships between oxidative stress and inflammation, consistent with established frameworks for understanding mediating variables in complex biological systems (33, 34).

Our multivariate analysis identified four independent predictors of NOX2 levels: HbA1c (β = 0.31, p < 0.001), BMI (β = 0.24, p = 0.003), diabetes duration (β = 0.19, p = 0.012), and family history (β = 0.16, p = 0.028). This model explains 38% of NOX2 variance (R² = 0.38, p < 0.001). These results position NOX2 as an integrator of metabolic (hyperglycemia, adiposity), temporal (disease duration), and genetic (family history) signals, suggesting that oxidative stress constitutes a central mechanism linking different risk factors to diabetic complications. This integrative vision has important implications for risk stratification and therapeutic personalization. NOX2 measurement could complement traditional markers (HbA1c, glucose, lipid profile) to better stratify individual risk and guide therapeutic intensification, particularly in patients with family history or poor glycemic control. A NOX2 threshold > 20 ng/mL could identify patients requiring enhanced surveillance and more aggressive therapeutic optimization.

Several factors specific to the Congolese population may modulate NOX2 activity and its pathophysiological impact, making our findings particularly relevant for understanding diabetes in African contexts: The urban population of Brazzaville is undergoing rapid dietary transition characterized by increased consumption of ultra-processed foods rich in trans fatty acids and refined sugars, decreased traditional diet rich in fiber (cassava, plantain, leafy vegetables), and higher dietary AGE (advanced glycation end-product) load from fried and grilled foods. This nutritional shift could exacerbate NOX2-dependent oxidative stress. Dietary AGEs directly activate NOX2 through RAGE (receptor for AGEs) signaling, creating a vicious cycle between hyperglycemia-induced AGE formation and oxidative stress amplification (35).

Studies in Central Africa have documented significant deficiencies in key antioxidants: vitamin E (35-45% prevalence of deficiency), selenium (28% below optimal levels), and zinc (40% subclinical deficiency in urban populations) (36). These deficiencies impair endogenous antioxidant defenses glutathione peroxidase requires selenium, superoxide dismutase requires zinc potentially amplifying the oxidative damage caused by NOX2 activation, creating a double burden of excessive ROS production coupled with inadequate antioxidant defenses. Indeed, earlier work in African populations has demonstrated strong associations between oxidative stress markers and metabolic syndrome features in diabetic patients (37), underscoring the clinical relevance of this oxidative-nutritional interplay.

The high unemployment rate in our cohort (52.4% vs 29.4% in controls) is accompanied by chronic psychosocial stress. Stress activates the hypothalamic-pituitary-adrenal axis, and cortisol has been shown to stimulate NOX2 expression and activity in immune and vascular cells. Chronic stress may therefore represent an underappreciated driver of oxidative stress in urban African populations undergoing rapid socioeconomic changes.

These airborne pollutants represent an often-overlooked source of oxidative stress, as they activate NOX2 in pulmonary macrophages and endothelial cells (38).

African populations exhibit distinct allelic frequencies for genes involved in oxidative metabolism. Polymorphisms in CYBB (NOX2), NCF1 (p47phox), and NCF2 (p67phox) may differ from Caucasian or Asian populations, potentially modulating basal NOX2 activity and response to hyperglycemia. This genetic diversity remains largely unexplored and justifies dedicated pharmacogenomic studies in African cohorts.

These contextual factors reinforce the importance of conducting research in diverse populations rather than extrapolating findings from Western studies. The interplay between genetic susceptibility, nutritional deficiencies, and environmental stressors may create a unique oxidative stress phenotype in sub-Saharan African patients with diabetes.

Pharmacological targeting of NOX2 represents a promising therapeutic strategy. Several selective NOX inhibitors have been developed, notably GKT137831 and VAS2870, which have shown efficacy in reducing oxidative stress and inflammation in murine diabetes models (31, 39, 40). While NOX2 predominates in immune cells and has been our focus here, it's worth noting that other members of the NOX family, particularly NOX5, also play important roles in vascular cells (41), and the broader family of NADPH oxidases contributes to various aspects of vascular pathology in diabetes (42). These molecules act by blocking NOX2 complex assembly or directly inhibiting catalytic subunit enzymatic activity. Although promising in preclinical research, none of these molecules has yet been approved for clinical use in humans, primarily due to persistent questions regarding their selectivity, pharmacokinetics, and potential adverse effects. 

Our data also highlight the need to integrate NOX2-dependent oxidative stress into early biomarker panels for diabetes and its cardiovascular complications. NOX2 measurement could complement traditional markers (HbA1c, glucose, lipid profile) to better stratify individual risk and guide therapeutic intensification, particularly in patients with family history or poor glycemic control. A NOX2 threshold > 20 ng/mL could identify patients requiring enhanced surveillance and more aggressive therapeutic optimization. This approach may be especially valuable in sub-Saharan African healthcare settings, where resources for comprehensive diabetes care are often limited and tools for predicting complications are urgently needed (43). Furthermore, given the sensitivity analysis showing that statin use did not significantly affect NOX2 levels (20.5 ± 5.0 vs 21.0 ± 5.3 ng/mL in non-users, p = 0.58), targeting oxidative stress through NOX-specific pathways may provide benefits beyond those achieved with conventional cardiovascular medications.

This study nevertheless has certain limitations, notably its cross-sectional nature does not allow establishment of strict causal relationships between NOX2 and observed metabolic alterations; analyses do not account for diurnal variations or potential nutritional influences; and finally, the sample, although representative, could be extended to other regions of Congo to strengthen result generalization. Despite these limitations, our observations provide novel data in sub-Saharan African populations, contributing to better understanding of oxidative stress’s role in diabetes.

## Conclusion

This study demonstrates that NOX2-mediated oxidative stress is intimately linked to inflammatory, apoptotic, and metabolic imbalances observed in Congolese patients with type 2 diabetes. Significant correlations between NOX2, HbA1c, glucose, IL-6, COX-2, and Caspase-3 confirm NOX2’s central role in T2D pathophysiology, as a mediator between inflammation and insulin resistance. The significant influence of family history on NOX2 levels reinforces the hypothesis of a genetic component in oxidative stress susceptibility, justifying pharmacogenomic studies in African populations where allelic frequencies for NOX2-related genes may differ substantially from other populations. The environmental and nutritional context of urban Congo characterized by rapid dietary transition, micronutrient deficiencies, and chronic psychosocial stress likely amplifies the oxidative burden in this population. Our results indicate that NOX2 could serve as a valuable biomarker for assessing T2D metabolic severity and potentially predicting risk of complications. After adjusting for glycemic control and disease duration, patients on insulin therapy showed significantly higher NOX2 levels, suggesting that NOX2 may help identify individuals with more severe disease requiring intensive therapeutic intervention. These findings pave the way for new therapeutic strategies targeting NOX2 and for using this biomarker as a prognostic indicator of diabetes metabolic severity. However, longitudinal and interventional studies are now necessary to evaluate NOX2’s predictive role in diabetic complication occurrence, to validate NOX2 as a therapeutic target, and to determine whether NOX inhibitors can reduce the burden of diabetic complications in sub-Saharan Africa, where healthcare resources for managing diabetes are often limited.

## Data Availability

The raw data supporting the conclusions of this article will be made available by the authors, without undue reservation.

## Glossary

ALT: alanine aminotransferase
AMPK: AMP-activated protein kinase
AST: aspartate aminotransferase
BMI: body mass index
COX-2: cyclooxygenase-2
CRP: C-reactive protein
CYBB: cytochrome b-245 beta chain
DPP-4i: dipeptidyl peptidase-4 inhibitors
GFR: glomerular filtration rate
GLP-1: glucagon-like peptide-1
HbA1c: glycated hemoglobin
HDL-C: high-density lipoprotein cholesterol
IBD: inflammatory bowel disease
IQR: interquartile range
IL-6: interleukin-6
LDL-C: low-density lipoprotein cholesterol
MODY: maturity-onset diabetes of the young
NCF1: neutrophil cytosolic factor 1
NCF2: neutrophil cytosolic factor 2
NF-κB: nuclear factor kappa B
NOX: NADPH oxidase
NOX2: NADPH oxidase 2
PKA: protein kinase A
ROS: reactive oxygen species
SD: standard deviation
T2D: type 2 diabetes
TINIA: turbidimetric inhibition immunoassay
TNF-α: tumor necrosis factor-alpha
WHO: World Health Organization
