# Supplementary material for: Role of NOX2 in the regulation of inflammatory and apoptotic pathways in congolese patients with type 2 diabetes in Brazzaville
Source: Front Endocrinol (Lausanne). 2026 Jan 13;16:1748586. doi: 10.3389/fendo.2025.1748586 (PMC12834810; doi:10.3389/fendo.2025.1748586)
Supplement: Supplementary file 1 [file DataSheet1.pdf]

SUPPLEMENTARY MATERIALS

Role of NOX2 in the Regulation of Inflammatory and Apoptotic Pathways in Congolese Patients with Type 2 Diabetes in Brazzaville

Feddercen Kelly Helga Mayassi[1,2], Charley Loumade Elenga-Bongo[1,7], Ghislain Loubano-Voumbi[1,3,5], Juste Brunhel Kaya Gondo[1,2,6], Jeancia Jordanie Mbemba Makele[1], Evariste Bouenizabila[1,4], Donatien Moukassa[1*]

[1]Doctoral Training, Faculty of Health Sciences, Marien Ngouabi University, Brazzaville, Republic of Congo

[2]Blanche Gomes Mother and Child Specialised Hospital Analytisis Laboratory, Brazzaville, Republic of Congo

[3]National Institute for Research in Health sciences, Brazzaville, Republic of Congo

[4]Teaching Hospital of Brazzaville, Brazzaville, Republic of Congo

[5]Dolisie General Hospital Analysis Laboratory, Brazzaville, Republic of Congo

[6]Institut Modulaire Participatif d'Utilité Locale Scientifique et Éducative, Monastère, France

[7] Adolph Sice Hospital General, Pointe noire, Republic of Congo

Correspondence : kellymayassi@gmail.com, ghisloubano@yahoo.fr

TABLE OF CONTENTS

Table S1. Multiple Linear Regression Analysis: Independent Predictors of NOX2 Levels Including Treatment Type (n = 143) 3

Table S2. NOX2 Levels According to Family History of Type 2 Diabetes 4

Table S3. Partial Correlation Analysis: NOX2 vs Inflammatory/Apoptotic Markers Controlling for HbA1c (n = 143) 6

Table S4. NOX2 Levels Stratified by Glycemic Control Status (n = 143) 6

Table S5. NOX2 Levels Stratified by Body Mass Index Category (n = 143)................... 7

SUPPLEMENTARY TABLE S1

These findings emphasize that NOX2-dependent oxidative stress is primarily driven by metabolic dysfunction (hyperglycemia, adiposity) and genetic predisposition, rather than by the specific antidiabetic medications used. This supports a treat-to-target approach focusing on achieving optimal HbA1c and weight management, regardless of the specific agents employed, as presented **Table S1**.

Table S1. Multiple Linear Regression Analysis: Independent Predictors of NOX2 Levels in Diabetic Patients (n = 143)

Variable	B	SE	β (standardized)	t	p-value	95% CI
**(Constant)**	-8.42	5.23	—	-1.61	0.110	[-18.76, 1.92]
**HbA1c (%)**	**+1.35**	**0.41**	**+0.31**	**+3.29**	**0.001**	**[+0.54, +2.16]**
**BMI (kg/m²)**	**+0.42**	**0.17**	**+0.24**	**+2.47**	**0.015**	**[+0.08, +0.76]**
**Diabetes duration (years)**	+0.15	0.08	+0.17	+1.88	0.062	[-0.01, +0.31]
**Family history (yes = 1)**	**+1.96**	**0.86**	**+0.19**	**+2.28**	**0.024**	**[+0.26, +3.66]**
**Age (years)**	+0.05	0.04	+0.10	+1.25	0.214	[-0.03, +0.13]
**Sex (Male = 1)**	+0.28	0.85	+0.03	+0.33	0.743	[-1.40, +1.96]
**Treatment type:** Insulin-based vs others	+1.24	1.28	+0.10	+0.97	0.334	[-1.29, +3.77]

Multiple R = 0.620, R^2 = 0.384, Adjusted R^2 = 0.347, Standard Error of Estimate = 4.23 ng/mL, **F(7, 135) = 10.32, p < 0.001. Abbreviations:** *β*, standardized regression coefficient; *SE*, standard error; *CI*, confidence interval; *VIF*, Variance Inflation Factor; *BMI*, body mass index; *HbA1c*, glycated hemoglobin.

SUPPLEMENTARY TABLE S2

Table S2. NOX2 Levels According to Family History of Type 2 Diabetes

The odds ratio of 1.38 suggests a 38% increased risk of developing T2D in individuals with first-degree family history compared to those without. Although this did not reach statistical significance in our sample size (p = 0.167), the confidence interval [0.87-2.18] indicates considerable uncertainty, and a larger study would likely demonstrate significant association. The point estimate is consistent with established literature showing 2- to 6-fold increased risk in first-degree relatives.

A. Prevalence of Family History

Group	With FH	Without FH	OR [95% CI]	p
	n (%)	n (%)		
T2D patients	68 (47.6%)	75 (52.4%)	1.38 [0.87-2.18]	0.167
Controls	54 (39.7%)	82 (60.3%)	Reference	

Chi-square test: $\chi^2(1) = 1.90$, $p = 0.167$

B. NOX2 Levels Stratified by Family History

Diabetic patients with family history exhibit significantly higher NOX2 levels (+2.27 ng/mL, p = 0.008), suggesting a genetic component in oxidative stress susceptibility. Importantly, this difference is not observed in healthy controls (p = 0.511), reinforcing the hypothesis of a gene-disease interaction i.e., genetic variants affecting NOX2 may only manifest clinically in the presence of metabolic stress.

The moderate effect size (Cohen's d = 0.44) indicates clinical relevance beyond statistical significance, as this represents an 11.5% elevation in oxidative stress that could accelerate complications.

Group	n	NOX2 (ng/mL)<br>Mean ± SD	95% CI	p-value
**T2D patients:**				
With family history	68	**22.00 ± 5.30**	[20.71, 23.29]	**0.008**
Without family history	75	**19.73 ± 4.87**	[18.61, 20.85]	
**Mean difference**		**+2.27 ng/mL** (11.5% higher)		
**Effect size**		Cohen's d = 0.44 (medium)		
**Controls:**				
With family history	54	5.10 ± 1.94	[4.57, 5.63]	0.511
Without family history	82	4.89 ± 1.69	[4.52, 5.26]	

Student's t-test (two-tailed). $p < 0.01$ considered statistically significant. **Abbreviations:** *FH*, Family History; *OR*, Odds Ratio; *T2D*, Type 2 diabetes; *CI*, confidence interval.

SUPPLEMENTARY TABLE S3

Table S3. Partial Correlation Analysis: NOX2 vs Inflammatory/Apoptotic Markers Controlling for HbA1c (n = 143)

Marker Pair	Zero-Order Correlation (Unadjusted)		Partial Correlation (Controlling for HbA1c)		Interpretation
	r_s	p	r_partial	p	
**NOX2 vs IL-6**	-0.212	< 0.001	-0.084	0.318	**Mediated by HbA1c**
**NOX2 vs COX-2**	-0.287	< 0.001	-0.189	0.024*	**Partially independent**
**NOX2 vs CRP**	+0.038	0.648	+0.021	0.806	**No association**
**NOX2 vs Caspase-3**	-0.098	0.243	-0.034	0.686	Mediated by HbA1c

* Remains statistically significant after adjustment (α = 0.05). **Abbreviations:** *r_s*, Spearman's rho; *AGE*, Advanced glycation end-products; *RAGE*, Receptor for AGE; *ROS*, Reactive oxygen species; *IL-6*, Interleukin-6; *COX-2*, Cyclooxygenase-2; *CRP*, C-reactive protein.

SUPPLEMENTARY TABLE S4

Table S4. NOX2 Levels Stratified by Glycemic Control Status (n = 143)

HbA1c Category	n	NOX2 (ng/mL)		Range	HbA1c (%)
		Mean ± SD	Median		Mean ± SD
**Well-controlled (< 7%)**	18	17.76 ± 4.12	17.05	[12.2-25.3]	6.39 ± 0.38
**Fair control (7-8.9%)**	58	20.28 ± 4.79	19.85	[12.3-29.8]	8.20 ± 0.52
**Poor control (≥ 9%)**	67	22.05 ± 5.27	21.50	[12.3-29.8]	10.08 ± 0.79

SUPPLEMENTARY TABLE S5

Table S5. NOX2 Levels Stratified by Body Mass Index Category (n = 143)

**BMI Category**	**n**	**NOX2 (ng/mL)**		**Range**	**BMI (kg/m^2)**
**(WHO classification)**		Mean ± SD	Median		Mean ± SD
**Normal weight (< 25)**	24	18.19 ± 4.21	17.65	[12.3-25.8]	23.39 ± 1.17
**Overweight (25-29.9)**	49	20.15 ± 4.89	19.70	[12.2-29.8]	27.16 ± 1.42
**Obesity (≥ 30)**	70	22.28 ± 5.19	21.95	[13.1-29.8]	32.62 ± 2.28

Abbreviations: *WHO*, World Health Organization; *BMI*, Body mass index; *TNF-α*, Tumor necrosis factor-alpha; *IL-6*, Interleukin-6; *HIF-1α*, Hypoxia-inducible factor-1 alpha; *ANCOVA*, Analysis of covariance; η^2, Eta-squared; *SE*, Standard error.
